# Congenital coralliform cataract is the predominant consequence of a recurrent mutation in the CRYGD gene

**DOI:** 10.1186/s13023-023-02816-0

**Published:** 2023-07-21

**Authors:** Kai-Jie Wang, Jue-Xue Wang, Jin-Da Wang, Meng Li, Jing-Shang Zhang, Ying-Yan Mao, Xiu-Hua Wan

**Affiliations:** 1 Beijing Tongren Eye Center, Beijing Tongren Hospital, Capital Medical University, Beijing Ophthalmology & Visual Sciences Key Lab, 100730 Beijing, China; 2 NO.1 Dong Jiao Min Xiang, 100730 Beijing, China

**Keywords:** Coralliform cataract, *CRYGD*, Mutation

## Abstract

**Background:**

Congenital cataract is a leading cause of treatable childhood blindness and both clinically and genetically heterogeneous. Among the already characterized phenotypes, coralliform cataract is a rare special form of congenital cataracts. Although previous studies had shown that mutations in the γD-crystallin (*CRYGD*) can result in congenital coralliform cataracts, no conclusive genotype-phenotype correlation might be drawn. Here we aimed to identify the spectrum and frequency of *CRYGD* gene mutations in congenital coralliform cataracts of Chinese origin.

**Methods:**

The medical records of 392 Chinese families with congenital cataracts were reviewed between January 2011 and December 2021. The families, clinically documented to have congenital coralliform cataracts, were screened for mutations in candidate *CRYGD* gene. The genomic DNA of all subjects was extracted from peripheral blood leukocytes. PCR amplified and direct sequencing were performed to identify the disease-causing mutation.

**Results:**

A total of 12 families with coralliform cataracts were recruited in this study in the past 10 years, accounting for 3.1% of the families with congenital cataracts. Of the 12 families, all affected individuals presented with bilateral non-progressive coralliform cataracts since birth, with the best-corrected Snellen visual acuities ranging from 20/200 to 20/25. A recurrent c.70 C > A (p. P24T) mutation in *CRYGD* was identified in 10 families (83.3%) with congenital cataract, which co-segregated with all affected individuals and was not observed in unaffected family members or ethnically matched normal controls.

**Conclusions:**

The coralliform cataract is characterized by being bilateral, non-progressive and present at birth. A recurrent p.P24T *CRYGD* mutation occurs independently in 83.3% of the Chinese families with congenital coralliform cataracts and most likely represents a mutational hot spot, which underscore the relations between coralliform cataract and p.P24T *CRYGD*.

## Background

Congenital cataract (CC), which refers to any opacification of the lens, is usually onset at birth or during one year after birth. It has been reported as one of the most common causes of blindness and severe visual impairment in childhood worldwide [1], with the overall prevalence of 0.63 to 9.74/10 000 children [2]. Wu et al. estimated the global CC prevalence to be 4.24/10,000, with the highest prevalence observed in Asia (7.43/10 000), followed by the USA (4.39/10 000), Europe (3.41/10 000) and Australia (2.23/10 000) [3]. A multicenter neonatal eye screening program in China reported CC accounted for 1.24% among 13,514 abnormal cases in 64 632 newborns [4].

The etiologies of CC are diverse and complicated. About one third of isolated congenital cataracts are genetically determined, of which autosomal dominant congenital cataract (ADCC) is the most common mode of inheritance [5, 6]. Clinical and genetic heterogeneity of congenital cataracts are well substantiated [6]. To date, at least 43 genes (http://cat-map.wustl.edu/) have been reported to be associated with various forms of isolated CC, including genes encoding crystallins, membrane proteins, transcription factors, cytoskeletal proteins and others [7]. Of the cataract mutations reported to date, about half of them involve mutations in crystallins, a quarter in connexins, and the rest divide among the other genes [1]. Crystallins play an important role in maintaining lens transparency, which constitute 90% of the lens proteins [8]. Mutations in major crystallin genes such as γ-crystallin (CRYG) in humans have been well documented. Among the already characterized phenotypes, coralliform cataract is a rare special form of congenital cataracts with a ‘coral-like’ pattern of opacity in the lens. Previous studies had shown that mutations in the *CRYGD* gene can result in congenital coralliform cataracts [9–14], although an insertional mutation in the connexin 46 had also been identified causing coralliform cataract in a Chinese family [15]. Therefore, it is appropriate to consider the *CRYGD* gene as the top list of functional candidates in congenital coralliform cataracts.

In this study, a total of 12 genetically unrelated families with autosomal dominant coralliform cataract were identified in the past 10 years. We performed the molecular analysis of the families with coralliform cataract to identify the *CRYGD* mutation spectrum and further analyze the genotype-phenotype correlations in Chinese families.

## Methods

### Subjects

This study was approved by the Medical Ethics Committee of Beijing Tongren Hospital and in accordance with the tenets of the Declaration of Helsinki. Twelve families with congenital coralliform cataracts were recruited at Beijing Tongren Hospital (Capital Medical University, Beijing, China), from January 2011 to December 2021. Both affected and unaffected individuals were subject to detailed ophthalmic examinations, including visual acuity, intraocular pressure, slit-lamp examinations; A-scan and B-scan ultrasonography; and fundus photochromy. No evidence of systemic abnormalities and other history of disease were examined in the probands. Unrelated control subjects were recruited from people who attended Beijing Tongren Hospital for eye examinations and aged older than 60 years without other eye diseases, except mild senile cataracts and mild refractive errors. Blood samples were collected from all participants after signing informed consent. Peripheral venous blood was collected for genomic DNA extraction using QIAamp DNA kit (Qiagen, Valencia, CA) according to the manufacturer’s protocol.

### Mutation analysis

PCR amplification was performed in the coding exons and splice sites of *CRYGD* gene (Genbank NM_006891.4) using primer pairs listed in Table 1. After purification, the PCR products were sequenced using an ABI3730 Automated Sequencer (PE Biosystems, Foster City, CA) to analyze the cosegregation of the genotype with the disease phenotype.

**Table 1:** Primer sequences for CRYGD

Amplicon	Forward Primers (5′→3′)	Reverse Primers (5′→3′)
1	CAACAAGCCCCGTGGTCTA	GGGTCCTGACTTGAGGATG
2	GCTTTTCTTCTCTTTTTATTTCTG	AAGAAAGACACAAGCAAATCAG

Forward and reverse primer sequences were provided for each amplicon of the CRYGD gene

The sequence of *CRYGD* in the probands was compared to the reference sequence (Genbank NM_006891.4) and potentially disease-causing variants were assessed for segregation with the disease in Sanger-sequenced affected and unaffected family members. The Genome Aggregation Database (gnomAD) v3.1.2 (https://gnomad.broadinstitute.org/) was used for variant analysis. Cat-Map (https://cat-map.wustl.edu/, accessed on 1 February 2023), an online chromosome map and reference database for cataract in humans, was used to search for previous variant descriptions and clinical associations.

## Results

### Clinical findings

**Fig. 1:**
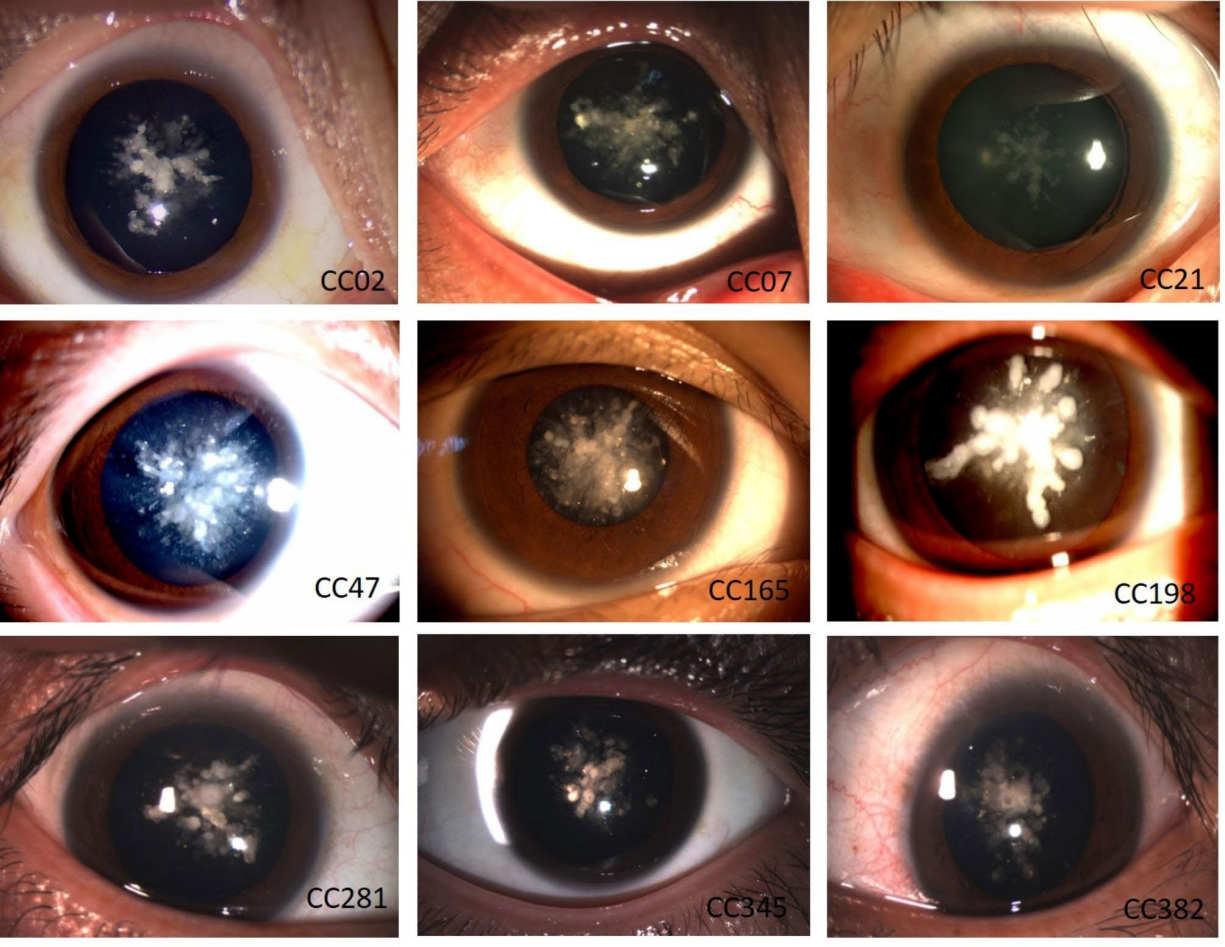
Slit lamp photographs of the probands identified p.P24T mutation. The photographs of the probands show coralliform shape opacification characterized by the white opaque involving the central portion of the lens

A total of 392 families with CC were identified in 2011–2021, twelve of them (3.1%) with coralliform cataracts. Of the 12 families, they were all from different ethnic groups in China, and all affected individuals had the same cataract phenotype, showing bilateral coralliform shape opacification characterized by the white opaque involving the central portion of the lens to a variable extent, with appearance resembling the coralliform shape (Fig. 1). A review of ophthalmic records indicated that bilateral and symmetrical cataracts were diagnosed at birth in all 12 families but were without progressive development of lens opacities, necessitating cataract extraction. The best-corrected Snellen visual acuities of the probands ranged from 20/200 to 20/25, with age-at-surgery ranging from 1 year to 17 years. The clinical characteristics of the probands in 12 families were summarized in Table 2.

**Table 2:** Clinical characteristics of the probands in our study

Family ID	Phenotype Description	Age of onset	BCVA (OD;OS)	Age at surgery(years)	Inheritance Pattern	Affected(N)/ Unaffected(N)
CC02 CC07 CC21 CC47 CC165 CC198 CC241* CC248 CC281 CC302* CC345 CC382	coralliform coralliform coralliform coralliform coralliform coralliform coralliform coralliform(cataract extraction) coralliform coralliform coralliform coralliform	SB SB SB SB SB SB SB SB SB SB SB SB	20/40; 20/50 20/40; 20/40 20/25; 20/25 20/200; 20/200 20/100; 20/200 20/100; 20/60 20/100; 20/100 20/200; 20/200 20/50; 20/40 20/200; 20/200 20/30; 20/30 20/30; 20/40	Not 17 Not 5 10 5 7 1 Not 7 Not Not	AD AD AD AD AD AD AD AD AD AD AD AD	7/10 7/9 9/13 5/11 6/7 4/10 4/9 5/7 3/4 2/5 3/4 5/6

SB, since birth; BCVA, best corrected visual acuity; AD, autosomal dominant; OD, oculus dexter; OS, oculus sinister

*No mutation found in CRYGD

### Mutation analysis

**Fig. 2:**
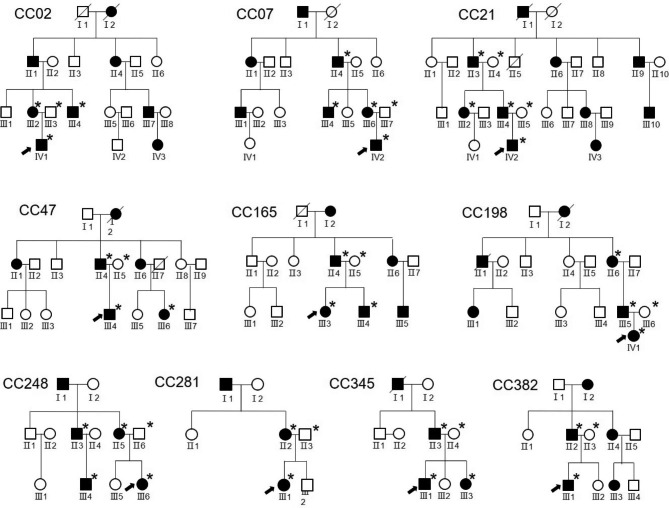
Pedigrees of the families identified mutations in this study. Squares and circles indicate males and females, respectively. Blackened symbols denote affected status. The proband is denoted by an arrow, and asterisks indicate participants enrolled in this study

**Fig. 3:**
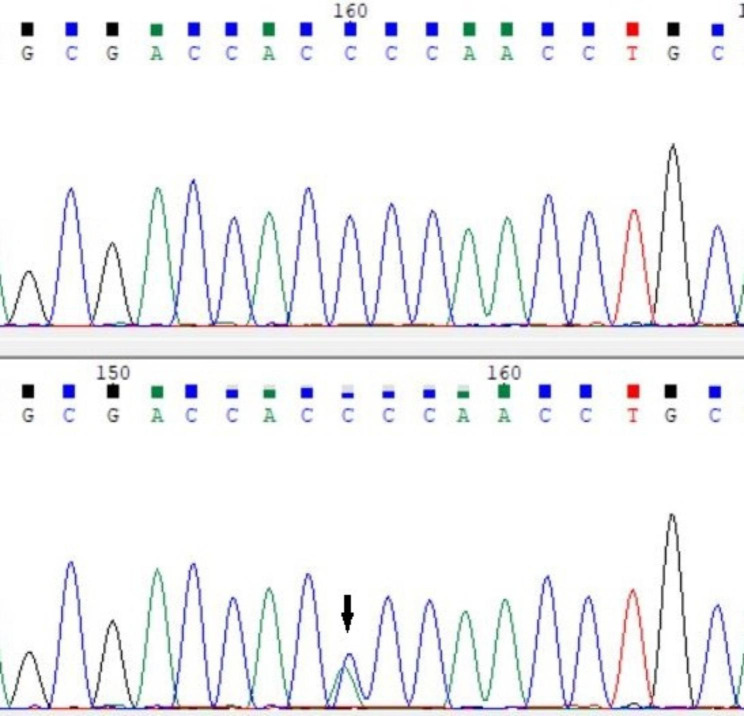
DNA sequence chromatograms. A single variant is observed at position 70 (C > A) as a C/A double peak (indicated by an arrow)

Direct sequencing of the entire coding region of *CRYGD* in 12 unrelated families with CC identified a recurrent c. 70 C > A mutation in 10 unrelated families (Fig. 2), which resulted in the substitution of proline at position 24 by threonine (p. P24T; Fig. 3). This variant was cosegregated with all affected individuals, and was not detected in any of the unaffected individuals or 110 normal controls. The variant had no or very low allele frequency in the gnomAD database, with a frequency of 0.003338% in South Asian population, but not detected in all the other populations of African/African-American, Latino/Admixed American, Ashkenazi Jewish, East Asian and European.

This study identified the p.P24T *CRYGD* mutation in 10 of 12 families from Chinese affected by coralliform cataracts, accounting for 83.3% of coralliform cataracts in this group of families. In contrast, no causative mutation in *CRYGD* gene was observed in family CC241 and CC302, which needed to be further investigated for the causative mutations.

## Discussion

In this study, we identified 12 families with bilateral and symmetrical congenital coralliform cataract in 392 CC families. To explore the relations between the *CRYGD* mutation and coralliform phenotype, the entire coding region of *CRYGD* in 12 unrelated Chinese families were sequenced. We identified a recurrent p.P24T mutation in 10 of 12 unrelated families, accounting for 83.3% of coralliform cataracts, and no other mutations in *CRYGD* were detected, which indicated that *CRYGD* might play an important role in the development of congenital coralliform cataract.

Crystallins are the predominant structural proteins in the human lens, comprised by two families with different characteristics: the α-crystallins, functioning as chaperones, and the βγ-crystallins, sharing the same structural unit “Greek key motif” [8]. As the smallest and simplest members of crystallins, γ-crystallins are mainly distributed in the nuclear region of the lens, and have two-domain structures with two Greek key motifs [16]. The solubility and stability of γD-crystallin is indispensable for the lens transparency. Mutation in *CRYGD* gene may destroy the solubility and stability of the crystallin proteins, subsequently reduce lens transparency causing CC.

Results of functional studies had shown that the p.P24T mutant protein had a significantly lower solubility compared with wild-type γD-crystallin [17]. Boatz et al. found that p.P24T variant aggregated under in vivo conditions with a native-like fold by a non-amyloid mechanism, which was considered to be the surface-mediated changes in protein–protein interactions [18]. Li et al. revealed that p.P24T mutant changed a pyrrole ring of the wild type into a hydrophilic structure, affecting the correct folding of the protein [19]. The findings presumed that the p.P24T might initiate aggregation or polymerization and result in the formation of CC.

Until now, at least 27 mutations in *CRYGD* gene, including p.P24T, have been reported to be associated with CC (http://cat-map.wustl.edu/). Different mutations presented with various phenotypes because of genotypic heterogeneity. For example, p.Y56X, p.R36P and p.R140X mutations were reported to be associated with nuclear cataract; the p.R77S was related with anterior polar coronary cataract; the p.R140X caused total cataract; p.W157X resulted in lamellar cataract [20–25]. In this study, we identified the p.P24T mutation in 83.3% of the Chinese families with coralliform cataract. Of interest, no mutation in *CRYGD* gene was observed in other two families, suggesting that the *CRYGD* might be the most common mutated gene in patients with coralliform cataract. This mutation had also been found independently in more than 20 pedigrees of different origin, as listed in Table 3. Among them, Yang et al. identified the p.P24T mutation in two Chinese families and compared the disease-associated haplotypes by analyzing microsatellites closely flanking the *CRYGD* gene. A different haplotype was found in the two families, strongly suggesting p.P24T may be a mutational hot spot but not a common founder [14]. In this study, our ten p.P24T-bearing families were from different ethnic groups in China. The distribution patterns together with reported haplotype data further supported this finding. However, the mechanism responsible for the increased mutation rate at position 24 needed to be further investigated.

**Table 3:** Summary of identified p.P24T mutation in the CRYGD

Mode of Inheritance	Morphology of Cataract	Other Phenotypes	Pedigrees	Origin	Reference
AD	lamellar	non-syndromic	1	Indian	[26]
AD	cerulean blue dot	non-syndromic	1	Moroccan	[27]
AD	silica-like nuclear	non-syndromic	1	Australia	[28]
AD	coralliform	non-syndromic	1	Chinese	[11]
AD	coralliform or axial	non-syndromic	1	Caucasian	[12]
AD	fasciculiform	non-syndromic	1	Chinese	[29]
AD	coralliform and cerulean	non-syndromic	2	Saudi	[13]
AD	coralliform	non-syndromic	2	Chinese	[14]
AD	aculeiform	non-syndromic	1	Indian	[30]
AD	coralliform	non-syndromic	1	Chinese	[31]
AD	coralliform	non-syndromic	1	Caucasian-American	[10]
AD	unknown		1	Australia	[32]
AD	coralliform	non-syndromic	2	Chinese	[33]
AD	coralliform	nystagmus	1	Chinese	[34]
AD	coralliform	non-syndromic	1	Chinese	[35]
AD	unkonwn	non-syndromic	1	Chinese	[24]
AD	unkonwn		1	Chinese	[36]
AD	coralliform	iris coloboma	1	Chinese	[19]
AD	Coralliform/ lamellar	non-syndromic	2	Chinese	[37]
AD	unknown		1	UK	[38]
AD	total	non-syndromic	1	Turkey	[39]
AD	coralliform	non-syndromic	2	Chinese	[9]

AD. autosomal dominant

p.P24T was also found to be responsible for several different phenotypes of CC except for coralliform cataract, e.g., lamellar cataract, cerulean cataract, the fasciculi form cataract and total cataract [9–14, 19, 24, 26–39]. Although there was variability in cataract phenotypes among the p.P24T-bearing families, coralliform cataract was the most common phenotype, and more importantly, all Chinese families including our ten families were involved coralliform cataract. Additionally, the clinical findings with regard to age of onset and progression were consistent in this study. These results underscored the close relations between non-progressive coralliform cataract and p.P24T *CRYGD*, at least in the Chinese population.

## Conclusions

In this study, 3.1% of 392 CC families had coralliform cataract, which was characterized by being bilateral, non-progressive and present at birth. The recurrent p.P24T *CRYGD* mutation was identified in 83.3% of the Chinese families with congenital coralliform cataract. Our results suggested that p.P24T mutation might be a mutational hot spot and closely related to the coralliform phenotype, which further provide evident for the molecular diagnosis and genetic counseling.

## Data Availability

All data generated and analyzed during the study are available in the published manuscript.
